# Identification and Validation of Immune-Related LncRNA Prognostic Signature for Lung Adenocarcinoma

**DOI:** 10.3389/fgene.2021.681277

**Published:** 2021-07-05

**Authors:** Guomin Wu, Qihao Wang, Ting Zhu, Linhai Fu, Zhupeng Li, Yuanlin Wu, Chu Zhang

**Affiliations:** ^1^School of Medicine, Shaoxing University, Shaoxing, China; ^2^Department of Thoracic Surgery, Shaoxing People’s Hospital, Shaoxing Hospital, Zhejiang University School of Medicine, Shaoxing, China; ^3^Department of Thoracic Surgery, The First Affiliated Hospital of Shaoxing University (Shaoxing People’s Hospital), Shaoxing, China

**Keywords:** lung adenocarcinoma, prognosis, lncRNA, immune infiltration, risk score

## Abstract

This study aimed to establish a prognostic risk model for lung adenocarcinoma (LUAD). We firstly divided 535 LUAD samples in TCGA-LUAD into high-, medium-, and low-immune infiltration groups by consensus clustering analysis according to immunological competence assessment by single-sample gene set enrichment analysis (ssGSEA). Profile of long non-coding RNAs (lncRNAs) in normal samples and LUAD samples in TCGA was used for a differential expression analysis in the high- and low-immune infiltration groups. A total of 1,570 immune-related differential lncRNAs in LUAD were obtained by intersecting the above results. Afterward, univariate COX regression analysis and multivariate stepwise COX regression analysis were conducted to screen prognosis-related lncRNAs, and an eight-immune-related-lncRNA prognostic signature was finally acquired (AL365181.2, AC012213.4, DRAIC, MRGPRG-AS1, AP002478.1, AC092168.2, FAM30A, and LINC02412). Kaplan–Meier analysis and ROC analysis indicated that the eight-lncRNA-based model was accurate to predict the prognosis of LUAD patients. Simultaneously, univariate COX regression analysis and multivariate COX regression analysis were undertaken on clinical features and risk scores. It was illustrated that the risk score was a prognostic factor independent from clinical features. Moreover, immune data of LUAD in the TIMER database were analyzed. The eight-immune-related-lncRNA prognostic signature was related to the infiltration of B cells, CD4+ T cells, and dendritic cells. GSEA enrichment analysis revealed significant differences in high- and low-risk groups in pathways like pentose phosphate pathway, ubiquitin mediated proteolysis, and P53 signaling pathway. This study helps to treat LUAD patients and explore molecules related to LUAD immune infiltration to deeply understand the specific mechanism.

## Introduction

Lung cancer has the highest morbidity and mortality among all cancers in the world (The Lancet, 2018). More than 50% of lung cancer patients have tumor metastasis when diagnosed, with a 5-year survival rate of only 5% (Siegel et al., 2020). According to histology, lung cancer is divided into small cell lung cancer and non-small cell lung cancer (NSCLC), and the latter accounts for about 85% of total lung cancer cases as a predominant subtype (Siegel et al., 2020). Lung adenocarcinoma (LUAD) accounts for 40% of all lung cancers, with a 5-year survival rate of only 15%, since patients are diagnosed when the cancer has been locally advanced and developed metastasis (Imielinski et al., 2012). To assist the diagnosis of early-stage LUAD and treat different patients with reasonable treatment regimens without wasting medical resources and delaying patient’s conditions, a suitable method is needed for predicting patient’s survival status.

Malignant phenotype of cancers is both influenced by cell characteristics itself and tumor microenvironment (TME) (Catalano et al., 2013). TME encompasses blood vessels around tumor cells, fibroblasts, stromal cells, immune cells, and different signaling molecules. Accumulating studies have suggested the correlation between the infiltration level of immune cells in TME and clinical outcomes. A study on colon carcinoma elaborated the impact of T cell infiltration on cancer recurrence rate (Mlecnik et al., 2011). Above all, tumor immune infiltration is crucial in cancer progression.

Long non-coding RNAs (lncRNAs) are a class of non-protein-coding RNAs more than 200 nt in length, and they directly or indirectly participate in regulating various life activities (Geisler and Coller, 2013). Moreover, they have been proved to be pertinent to immune infiltration of tumor tissue and to regulate cancer progression through TME. For example, downregulated lncRNA SATB2-AS1 hampers tumor metastasis *via* regulating SATB2 in colorectal cancer, and lncRNA SATB2-AS1 expression negatively correlates immune infiltration (Xu et al., 2019). LncRNA NEAT1 interacts with DNMT1 to affect lung cancer progression and immune cell infiltration *via* cGAS/STING and p53 pathways (Ma et al., 2020). Knocking down lncRNA NEAT1 constrains the viability, proliferation, and invasion of lung cancer cells, while facilitating the infiltration of cytotoxic T cells in tumor tissue (Ma et al., 2020). Therefore, lncRNAs related to tumor immune infiltration can work as potential prognostic biomarkers.

Lung adenocarcinoma patients in the TCGA-LUAD dataset were grouped by immune viability in TME. Then, immune-related differentially expressed lncRNAs (DElncRNAs) were obtained by differential expression analysis. The screened DElncRNAs were applied to establish a risk score model. The model could predict patient’s prognosis using the expression level of lncRNA marker to assist with choosing suitable therapeutic plans. The obtained lncRNAs can serve as researching objects in the subsequent exploration of immune infiltration regulation on LUAD to deeply understand immune escape mechanism and to dig novel therapeutic targets.

## Materials and Methods

### Patient’s Samples Grouped by Immunological Competence

Long non-coding RNA count data, mRNA FPKM data (normal: 59, tumor: 535), and corresponding clinical information were accessed from The Cancer Genome Atlas (TCGA)-LUAD in the TCGA database^1^. Single-sample gene set enrichment analysis (ssGSEA) was conducted on mRNA expression profile in cancer tissue of LUAD patients with R package “GSVA.” Cancer tissue enrichment was scored by a gene set based on prognosis-related immune microenvironment markers obtained by Bindea et al. (2013). Next, to classify cancer tissue by immunological competences, the *k*-means algorithm in R package “ConsensusClusterPlus” and the two-phase sampling method were applied, with sampling 80% each time and 1000 times of repetition. A consensus clustering analysis was performed on cancer tissue samples according to scores of ssGSEA to obtain the most stable group.

### Validation of Grouping Efficiency

Profiles of mRNA expression of cancer tissue samples were analyzed using R package “ESTIMATE” to score each sample. The immune score, stromal score, ESTIMATE score, and tumor purity of each sample were acquired. Human lymphocyte antigen (HLA) family genes and CD274 gene expression of cancer tissue in each group were analyzed by using R package “ggpubr.” The efficiency of cancer tissue grouping was detected with the above results.

### Screening of Immune-Related lncRNA in LUAD

A differential expression analysis (|logFC|>1, FDR<0.01) was carried out on lncRNA expression profiles in normal tissue and cancer tissue in TCGA-LUAD using R package “edgeR.” DElncRNAs related to LUAD occurrence were obtained. Differential expression analysis (|logFC|>1, FDR<0.01) was undertaken on lncRNAs of cancer tissue in line with groups classified by immunological competence, and immune-related differentially expressed lncRNAs were obtained. DElncRNAs in the above two groups were intersected to acquire final immune-related DElncRNAs in LUAD.

### Construction of Immune-Related lncRNA Prognostic Signature

Samples in TCGA-LUAD with a follow-up time less than 30 days were removed. Univariate COX regression analysis (*p* < 0.001) was conducted on immune-related DElncRNAs in LUAD using R package “survival” to screen lncRNAs that were correlated with LUAD prognosis. Then, multivariate COX regression analysis was undertaken through stepwise regression using dual-choice assay. The model with the smallest Akaike Information Criterion (AIC) was chosen as the immune-related lncRNA prognostic model. Patient’s risk score was calculated by the prognostic model, with the expression level of lncRNA as the risk factor. The higher the risk score, the poorer the predicted prognosis of LUAD patients. The formula is $R\,i\,s\,k\,s\,c\,o\,r\,e=\sum_{i=1}^{n}\left(C\,o\,e\,f\,i^{*}\,\chi \,i\right)$ (*Coef*_*i*_: risk coefficient, *χ_*i*_*: gene expression level standardized by *Z*-score). Clinical information of samples used in the analysis is shown in Supplementary Table 1.

### Validation of the Accuracy of the Prognostic Model

To validate the predictive accuracy of the immune-related lncRNA prognostic signature, risk score of patients in TCGA-LUAD was obtained from the prognostic model. Patients were divided into high- and low-risk groups based on the median score. Kaplan–Meier survival analysis was undertaken on two groups using R package “survival” to compare the survival rate. Afterward, R package “survivalROC” was employed to draw the receiver operating characteristic (ROC) curves of 3-year overall survival (OS) and 5-year OS. Thereafter, the area under curve (AUC) was calculated. The risk score distribution and survival status of patients were plotted to determine the accuracy of the model. At the same time, a heatmap of the expression level of lncRNAs in the prognostic model in cancer tissue of patients in high- and low-risk groups was drawn.

### Validation of the Independence of the Prognostic Model

To detect whether the risk score of patients evaluated by the immune-related lncRNA prognostic signature is independent of clinical features as a prognostic factor of LUAD, univariate and multivariate COX regression analyses were performed combining with risk scores and clinical traits (age, gender, T stage, N stage, and clinical stage). Next, R package “survivalROC” was applied to draw time-dependent ROC curves of the above prognostic factors, and AUCs were calculated, respectively.

### Correlation Analysis Between the Risk Score of the Prognostic Model and Infiltration of Each Immune Cell Subgroup in LUAD Samples

Immune infiltration data of B cells, CD4+ T cells, CD8+ T cells, dendritic cells, macrophages, and neutrophils were downloaded from the TIMER database^2^. The correlation between the risk score and infiltration of each immune cell subgroup was detected, respectively, by using Pearson correlation analysis.

### GSEA Enrichment Analysis in the High- and Low-Risk Groups

Pathway differences between the high- and low-risk groups were investigated by GSEA enrichment analysis (Mootha et al., 2003; Subramanian et al., 2005). mRNA expression microarray in the high- and low-risk groups in TCGA-LUAD was taken as the expression dataset. c2.cp.kegg.v7.4.symbols.gmt was selected as enrichment analysis gene set to run GSEA software. Displacement test times were set as 1000 and gene set with FDR < 0.25 was considered significant enrichment.

## Results

### LUAD Patients Are Grouped According to Immunological Competence

**FIGURE 1:**
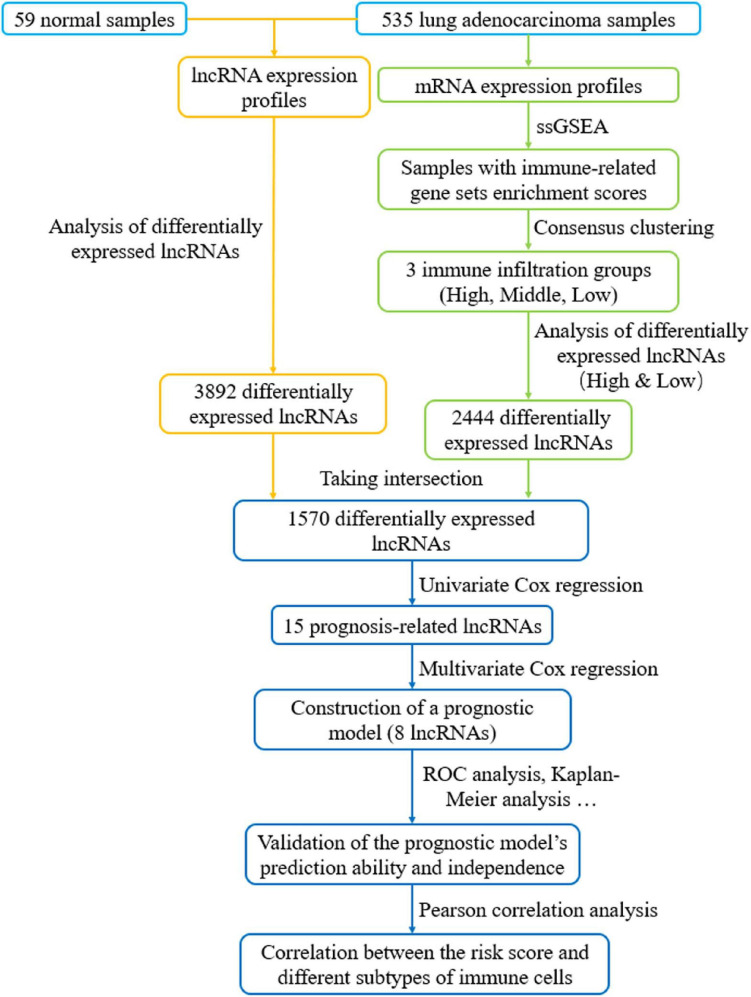
Flow chart of constructing a prognostic signature based on eight immune-related lncRNAs. LncRNAs related to immune infiltration and cancer occurrence were screened to build an eight-lncRNA prognostic signature. The predictive accuracy and independence of the signature in prognosis of LUAD were testified, and correlation between the signature-based risk score and abundance of several immune infiltrates was detected.

**FIGURE 2:**
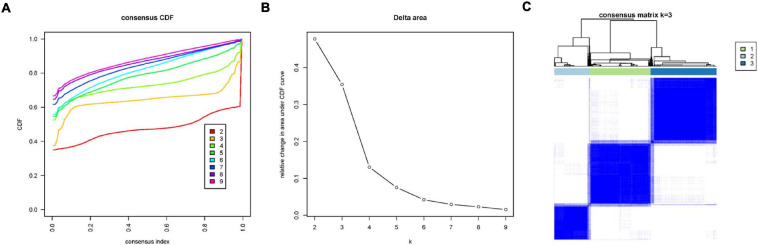
Consensus clustering analysis of LUAD tissue samples in TCGA-LUAD according to immune infiltration degree. **(A)** Consensus clustering cumulative distribution function (CDF) diagram with a tissue clustering number from *k* = 2 to *k* = 9. **(B)** Delta area plot of consensus clustering analysis of LUAD tissue, and the relative changes in CDF AUC in different *k*-values compared with that in *k*-1. *k*-value was finally chosen as 3, combining a series of analysis results. **(C)** Heatmap of sample consensus after grouping.

**FIGURE 3:**
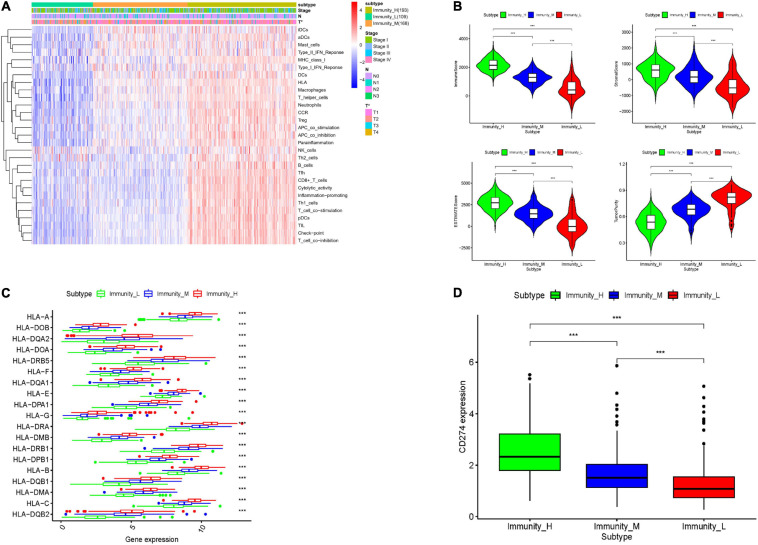
Validation of grouping effect of LUAD tissue samples. **(A)** Samples were divided into Immunity_H, Immunity_M, and Immunity_L groups according to immunological competence from high to low. Heatmap shows ssGSEA enrichment value of each immune-related gene set of LUAD tissue in each group, and tumor purity, immune score, stromal score, and ESTIMATE score obtained by “ESTIMATE” analysis. **(B)** Violin plots of tumor purity, immune score, stromal score, and ESTIMATE score of three groups of LUAD tissue. **(C)** Boxplots of the expression of each HLA family gene in LUAD tissue in three groups. **(D)** Boxplots of the expression of CD274 gene in LUAD tissue in three groups. *** represents *p* < 0.001 (difference between groups is highly statistically significant).

To explore the immune infiltration and prognosis of LUAD, this study established an immune-related LUAD prognostic model by bioinformatics method (Figure 1). mRNA expression profile of cancer tissue samples in TCGA-LUAD was analyzed *via* ssGSEA, and the immunological competence of each cancer tissue sample was scored. Afterward, all cancer tissue samples were divided into three groups by consensus clustering analysis according to the score: high-immune infiltration group (Immunity_H, 217 samples), medium-immune infiltration group (Immunity_M, 200 samples), and low-immune infiltration group (Immunity_L, 118 samples) (Figures 2A–C). Chi-square test unveiled remarkable differences in T stage distribution of patients in different immune infiltration groups (*p* < 0.05). To verify the effect of grouping, ESTIMATE algorithm was applied to score each sample in three groups, and it was found that the immune cell infiltration in cancer tissue was positively correlated with the immune score, stromal score, and ESTIMATE score, while it was negatively correlated with tumor purity (Figure 3A). It was also displayed that differences in infiltration of immune cells in varying immune groups were highly statistically notable (*p* < 0.01) (Figure 3B). Besides, the expression of HLA family genes and CD274 gene of cancer tissue sample in three groups was analyzed. The expression of immune-related genes elevated as immunological competence increased. It was also illustrated that differences in the expression of these genes in three groups were highly statistically significant (*p* < 0.01) (Figures 3C,D). All in all, three groups of LUAD subtypes divided by immunological competence in this study had different degrees of immune infiltration.

### Screening of Immune-Related Differential lncRNAs in LUAD

**FIGURE 4:**
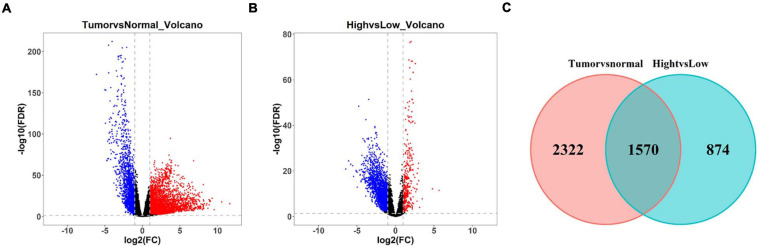
Screening of differentially expressed lncRNAs related to immune infiltration in LUAD. **(A)** Volcano plot of differential expression analysis of lncRNAs in LUAD tissue and normal tissue. Blue dots represent downregulated lncRNAs and red dots represent upregulated lncRNAs (| logFC| > 1, FDR < 0.01). **(B)** Volcano plot of differential expression analysis of lncRNAs in LUAD tissue in Immunity_H and Immunity_L groups. Blue dots represent downregulated lncRNAs and red dots represent upregulated lncRNAs (| logFC| > 1, FDR < 0.01). **(C)** Venn diagram of differential lncRNAs related to LUAD occurrence in panel **(A)** (red circle) and differential lncRNAs related to immune infiltration in panel **(B)** (green circle).

Differential expression analysis was undertaken on LUAD tissue and normal tissue. A total of 3,202 differentially upregulated lncRNAs and 690 downregulated lncRNAs in LUAD tumor tissue were obtained (| logFC| > 1, FDR < 0.01) (Figure 4A). Thereafter, differential expression analysis was performed on lncRNAs in the Immunity_H group and Immunity_L group. A total of 397 upregulated lncRNAs and 2,047 downregulated lncRNAs in the Immunity_H group were obtained (| logFC| > 1, FDR < 0.01) (Figure 4B). The duplicate lncRNAs in two groups were chosen to acquire 1,570 immune-related differential lncRNAs in LUAD (Figure 4C), and these lncRNAs could be used to screen LUAD prognostic signature in the following experiments.

### Construction of the Immune-Related lncRNA Prognostic Signature and Validation of Its Accuracy

**FIGURE 5:**
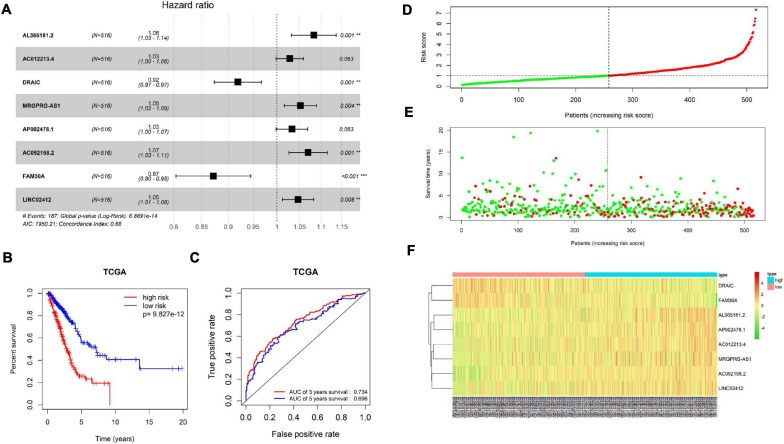
Construction and validation of the eight-immune-related-lncRNA prognostic signature. **(A)** Screening and construction of an eight-immune-related-lncRNA prognostic signature by multivariate COX regression analysis. Forest plot shows hazard ratio (HR) and *p*-value of each lncRNA in the model. ** represents *p* < 0.01, *** represents *p* < 0.001. **(B)** Survival curves obtained by Kaplan–Meier survival analysis in high-risk and low-risk groups divided by the median risk score. **(C)** ROC curves and corresponding AUCs of 3-year survival and 5-year survival predicted by the prognostic model. **(D)** Distribution diagram of risk score of LUAD patients in TCGA-LUAD. The horizontal dotted line indicates the median risk score of all patients. **(E)** Distribution diagram of survival status of LUAD patients. Patient’s risk score is presented from low to high by left to right. The vertical dotted line represents the patients with a median risk score. Green dots represent survival and red dots represent death. **(F)** Heatmap of the expression of eight immune-related lncRNAs in cancer tissue of LUAD patients in high- and low-risk groups.

Patient’s clinical data in TCGA-LUAD were obtained, and samples with less than 30 days of follow-up were removed. Then, univariate COX regression analysis was conducted on 1,570 immune-related differential lncRNAs in LUAD, and 15 lncRNAs highly significantly correlating patient’s prognosis were obtained (*p* < 0.001) (Supplementary Table 2). Next, multivariate COX regression analysis was applied on the 15 lncRNAs to acquire lncRNAs (AL365181.2, AC012213.4, DRAIC, MRGPRG-AS1, AP002478.1, AC092168.2, FAM30A, and LINC02412), and the expression of the eight lncRNAs was taken as prognostic factors of the risk score model (Figure 5A and Supplementary Table 3). The model predicted patient’s prognosis by calculating risk scores. The higher the risk score, the poorer the prognosis. LUAD patients in TCGA-LUAD were divided into high-risk group and low-risk group according to the median risk score of all tumor samples (Figure 5D). Kaplan–Meier survival analysis was carried out on patients in two groups, and it was found that the survival rate of patients in the high-risk group was significantly lower than that in the low-risk group (*p* < 0.001) (Figure 5B). ROC curves of 3-year and 5-year survival predicted by the prognostic model were drawn, and AUCs were calculated (3-year AUC: 0.734, 5-year AUC: 0.696). These indicated that the prognostic model could predict 3-year survival rate and 5-year survival rate of LUAD patients to a certain extent, and the accuracy and sensitivity in predicting 3-year survival rate were relatively high (Figure 5C). Meanwhile, it was found through statistical analysis that the mortality of patients in the high-risk group was higher than that in the low-risk group (Figure 5E). Moreover, compared with the expression in the low-risk group, the expression of lncRNAs (AL365181.2, AC012213.4, MRGPRG-AS1, AP002478.1, AC092168.2, and LINC02412) that were not conductive to positive prognosis in the high-risk group was higher, while the expression of the other two lncRNAs (DRAIC and FAM30A) that were conducive to positive prognosis in the high-risk group was lower (Figure 5F). The above results indicated that the constructed eight-immune-related-lncRNA prognostic signature could accurately predict LUAD patient’s prognosis.

### Examination of the Independence of the Eight-Immune-Related-lncRNA Prognostic Signature

**FIGURE 6:**
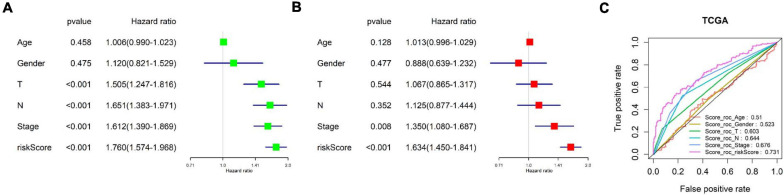
Examination of the independence of the risk score predicted by the prognostic model. **(A)** Univariate COX regression analysis was conducted on clinical traits (age, gender, T stage, N stage, and clinical stage) and the risk score. The forest plot shows HR and *p*-value of each prognostic factor. **(B)** Multivariate COX regression analysis was performed on clinical traits and the risk score. **(C)** ROC curves and AUCs of 3-year survival of each prognostic factor.

Since many clinical traits are related to prognosis of cancer, we focused on detecting the independence of the eight-immune-related-lncRNA prognostic signature. A univariate COX regression analysis was conducted on clinical traits (age, gender, T stage, N stage, and clinical stage) and the risk score calculated by the prognostic model. It was exhibited that T stage, N stage, clinical stage, and the risk score were significantly correlated with LUAD patient’s prognosis (*p* < 0.001) (Figure 6A). Afterward, a multivariate COX regression analysis was performed on clinical traits and the risk score. It was presented that only clinical stage and the risk score were remarkably related to LUAD patient’s prognosis (*p* < 0.05), and only the risk score displayed a highly significant correlation (*p* < 0.001) (Figure 6B). In addition, the AUC value for 3-year survival of the risk score was the highest (0.731) among the AUC values of other factors (Figure 6C). In conclusion, the eight-immune-related-lncRNA prognostic signature was an independent prognostic factor of LUAD prognosis.

### Analysis of the Correlation Between the Eight-Immune-Related-lncRNA Prognostic Signature and the Infiltration of Each Immune Cell Subgroup in LUAD Samples

**FIGURE 7:**
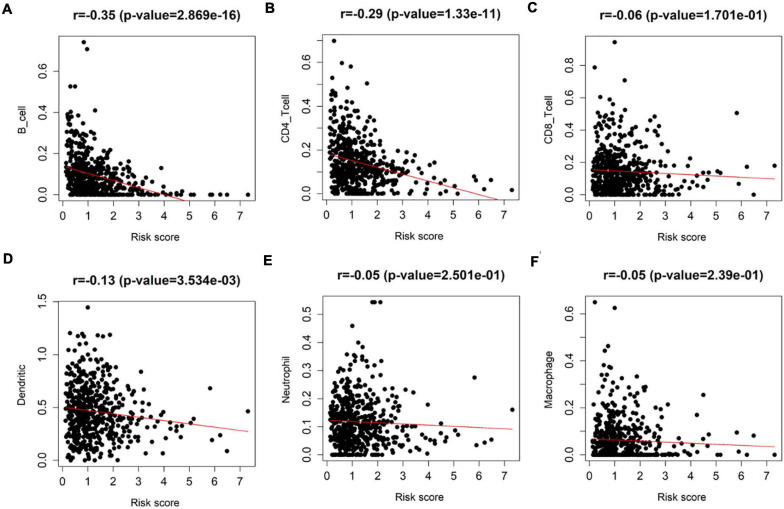
Detection of the correlation between the eight-immune-related-lncRNA prognostic signature and each immune cell subgroup. Pearson correlation analysis was undertaken to detect the correlation between the risk score of LUAD patients predicted by the model and the infiltration of **(A)** immune B cells, **(B)** CD4+ T cells, **(C)** CD8+ T cells, **(D)** dendritic cells, **(E)** neutrophils, and **(F)** macrophages in patient’s cancer tissue.

Since all factors in the prognostic model were related to immune infiltration, to explore the correlation between the eight-immune-related-lncRNA prognostic signature and immune infiltration, Pearson correlation analysis was performed on the infiltration of varying immune cell subgroups in LUAD samples and the eight-immune-related-lncRNA prognostic signature using data in the TIMER database. It was revealed that the risk score was negatively correlated with the infiltration of B cells, CD4+ T cells, and dendritic cells (*p* < 0.01) (Figures 7A–F). Thus, it was suggested that the risk score predicted for patients by the eight-immune-related-lncRNA prognostic signature could reflect the infiltration of B cells, CD4+ T cells, and dendritic cells.

### GSEA Enrichment Analysis in the High- and Low-Risk Groups

**FIGURE 8:**
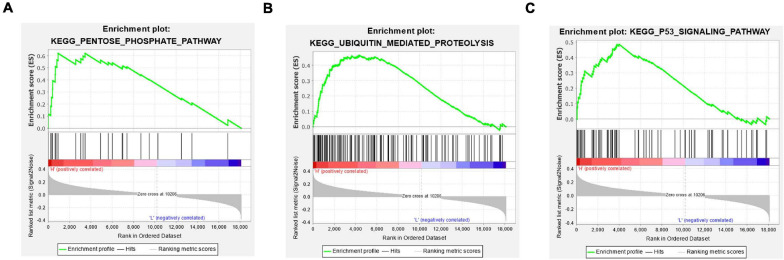
GSEA enrichment analysis in the high- and low-risk groups. **(A–C)** Patients in the high- and low-risk groups showed marked differences in pentose phosphate pathway, ubiquitin mediated proteolysis, and P53 signaling pathway signaling pathways, respectively.

The above analyses discovered significant correlation between the prognostic characteristics of eight immune-related lncRNAs and immune cell infiltration. In a bid to investigate the specific signaling pathways affected by these eight lncRNAs, GSEA enrichment analysis was undertaken in the high- and low-risk groups in this study. The results showed significant differences in signaling pathways (pentose phosphate pathway, ubiquitin mediated proteolysis, and P53 signaling pathway) between the high- and low-risk groups (Figures 8A–C). These pathways were found to be vital in tumor progression and directly or indirectly modulate immune characteristics of cancers (Liu et al., 2018; Giacomini et al., 2020; Li and Song, 2020). Above all, eight immune-related lncRNA prognostic characteristics were closely associated with some important cancer-related signaling pathways.

## Discussion

In this study, consensus clustering analysis was performed on patients in TCGA-LUAD for analyzing immunological competence. The immune infiltration of the patients was determined by using ESTIMATE algorithm plus the expression level of HLA family genes and the CD274 gene. The CD274 gene, which plays an important role in the immune escape of cancer cells, encodes programmed death ligand-1 (PD-L1) expressing in many cancer cells and immune cells. Expression of PD-L1 in cancer cells binds to programmed death receptor-1 (PD-1) in cytotoxic T cells to inhibit proliferation, migration, and secretion of cytotoxic substances, so as to impair the activity of cytotoxic T cells and thus prevent the tumor cells from being killed (Herbst et al., 2014; Kim and Chen, 2016). HLA gene clusters encode the major histocompatibility complex (MHC) of human, which can be divided into HLA class I and HLA class II. HLA is involved in antigen processing and presentation. Its abnormal expression is thought to be related to malignant transformation and immune escape of cells. For example, loss of HLA class I molecule expression will affect the interaction between cancer cells and T cells, leading to the ability of cancer cells to evade immune surveillance, and patients with loss of HLA class I molecule expression tend to have a shorter survival time (Garrido et al., 2017). Among the HLA genes, HLA-G gene expression was found to be upregulated when cells develop malignant phenotype, and it is identified to be associated with cancer inflammation, expressed in both cancer cells and immune cells, especially in CD68+ macrophages and CD8+ T cells infiltrating cancer tissue (Rouas-Freiss et al., 2003). There is an important relationship between immune infiltration and prognosis, suggesting that immune-related molecules can be used as prognostic markers in cancer patients.

Then, after screening and obtaining immune-related DElncRNAs in this study, univariate COX regression analysis combined with multivariate COX regression analysis was used to construct an eight-immune-related-lncRNA prognostic signature. Among the eight risk factors of the risk score model, AL365181.2, AC012213.4, MRGPRG-AS1, AC092168.2, and LINC02412 have been rarely studied, and a few studies were conducted on the remaining three lncRNAs. By constructing ceRNA networks in hepatocellular carcinoma, AP002478.1 was screened for many times as a potential prognostic marker (Liao X. et al., 2019; Yue et al., 2019). In hepatitis virus-positive hepatocellular carcinoma (Huang et al., 2019) and *Helicobacter pylori*-positive gastric cancer (Liu et al., 2019), AP002478.1 was also screened and found to be a prognostic marker to participate in the construction of prognostic models. In addition, AP002478.1 in the models constructed in the above studies is a negative prognostic factor, which is consistent with the results of this study. However, the role of AP002478.1 in LUAD has not been discussed in relevant studies, and the specific mechanism of its influence on cancer prognosis is still unknown.

Long non-coding RNA FAM30A was considered as a positive prognostic factor for LUAD in this study. In past studies, FAM30A was found to be correlated with immunity. For example, the expression level of FAM30A in periodontitis was positively related to the proportion of plasma cells in inflammatory tissue (Wu et al., 2020). In a study on the body’s response to vaccination and the protective mechanism induced by vaccines, the expression level of FAM30A was positively correlated with the antibody titer level after vaccination. Compared with other immune cells, the expression level of FAM30A was higher in B cells (de Lima et al., 2019). Therefore, FAM30A is closely immune-related. In the end of this study, the correlation between prognostic risk score and infiltration of multiple immune cell subgroups was analyzed. The eight-immune-related-lncRNA prognostic signature was significantly negatively correlated with B cells, CD4+ T cells, and dendritic cells, expanding subsequent research directions for FAM30A, AP002478.1, and other lncRNAs.

Studies on lncRNA DRAIC are more abundant, and there have been relevant functional studies. For example, it was found in gastric cancer cells that DRAIC can promote the degradation of NFRKB and inhibit the proliferation and metastasis of gastric cancer cells by blocking NFRKB de-ubiquitination mediated by UCHL5 (Zhang et al., 2020). In prostate cancer, DRAIC binds to IKK subunits to block interunit binding and inhibit NF-κB activation, thereby inhibiting invasion and proliferation of cancer cells (Saha et al., 2020). The above results indicate that DRAIC is a prognostic factor for cancer patients, which is the same as the analysis results in this study. In nasopharyngeal carcinoma studies, it was found that DRAIC can sponge miR-122 to promote the expression of SATB1, thus promoting the proliferation, migration, and invasion of nasopharyngeal carcinoma cells (Liao B. et al., 2019). DRAIC has diverse functions in different cancers. Another study also showed that DRAIC has the function of regulating autophagy (Tiessen et al., 2019). The above studies and this study can guide follow-up research directions of the screened eight prognostic lncRNAs, so as to conduct in-depth research on corresponding specific regulatory mechanism of the immune microenvironment of LUAD.

Viewed *in toto*, in this study, the mRNA expression profile of TCGA-LUAD was analyzed by ssGSEA and the immunological competence of the samples was scored to divide samples into three groups by consensus clustering analysis. Then, the differential expression analysis on the lncRNA profile of the samples in the high- and low-immune infiltration groups was performed. DElncRNAs obtained were intersected with the differential lncRNAs screened from the differential expression analysis in the normal samples and LUAD samples to obtain immune-related DElncRNAs in LUAD. Eventually, an eight-immune-related-lncRNA prognostic signature was obtained and a risk score model was established. Additionally, the risk score evaluated by this model was significantly negatively correlated with infiltration of B cells, CD4+ T cells, and dendritic cells. GSEA enrichment analysis discovered that patients in the high- and low-risk groups had marked differences in cancer-related signaling pathways. The risk score can reflect the patient’s immune microenvironment to some extent, thereby helping patients choose appropriate treatment. However, the accuracy of the risk score model on the prediction of 5-year survival rate is not high; therefore, more data and experimental basis are still needed to improve the model. Besides, this study did not use independent validation sets to test the accuracy of the risk score model; hence, the universal applicability of the model has not been verified.

## Data Availability Statement

The original contributions presented in the study are included in the article/Supplementary Material, further inquiries can be directed to the corresponding author/s.

## Author Contributions

GW contributed to the study design. CZ conducted the literature search, revised the article, and gave the final approval of the version to be submitted. QW and TZ acquired the data. LF and ZL wrote the article. YW performed the data analysis. ZL drafted the manuscript.

## Conflict of Interest

The authors declare that the research was conducted in the absence of any commercial or financial relationships that could be construed as a potential conflict of interest.

## Footnotes

1 https://portal.gdc.cancer.gov/

2 https://cistrome.shinyapps.io/timer/
